# Intestinal Metaplasia Associated with Symptoms of Dyspepsia

**DOI:** 10.21203/rs.3.rs-3335631/v1

**Published:** 2023-10-03

**Authors:** Omar Viramontes, Dalia Martinez, Ma Somsouk

**Affiliations:** University of California, Davis; University of California, San Francisco; University of California, San Francisco

**Keywords:** Gastric Intestinal Metaplasia (GIM), dyspepsia, Helicobacter pylori (HP), chronic gastritis (CG), chronic active gastritis (CAG), iron deficiency anemia (IDA), social safety-net hospital, upper endoscopy

## Abstract

**Background:**

Peptic ulcer disease (PUD) and Helicobacter pylori (HP) are associated with dyspepsia, but the role of gastric intestinal metaplasia (IM) has not been described. The objective of this study is to examine the association between gastric IM and dyspepsia.

**Methods:**

We developed a cohort of consecutive patients referred to gastroenterology between Jan 2019 and July 2020 for dyspepsia and iron deficiency anemia (IDA) and completed an upper endoscopy with biopsies in a safety-net health system. The primary outcome was the prevalence of gastric IM in patients with dyspepsia compared to IDA. Secondary outcomes included prevalence of HP, chronic gastritis (CG) and chronic active gastritis (CAG) in the dyspepsia and IDA groups. A multivariable analysis was performed to assess the independent association between gastric IM and dyspepsia

**Results:**

Compared to the IDA cohort (n = 366), patients with dyspepsia (n = 349) were more likely to be female (65% vs. 47%, p < 0.01), harbor gastric IM (20.3% vs. 14.2%, p = 0.03), and less likely to have CAG (12.0% vs. 26.5%, p < 0.01) or HP (10.9% vs. 21.3%, p < 0.01). After adjusting for pathological findings, race, ethnicity, gender and age, the association strengthened between IM and dyspepsia (adj OR 1.81 from OR 1.54, 95% CI 1.19–2.76, p < 0.01).

**Conclusions:**

We observed a significant relationship between the presence of gastric IM and dyspepsia symptoms, which increased after adjusting for confounding factors. Future studies should verify the relationship between IM and dyspepsia, the effect of IM regression, and possible mediators of gastric IM on symptoms.

## BACKGROUND

Existing evidence exist for the role of peptic ulcer disease (PUD) and Helicobacter pylori (HP) and dyspepsia; however, the role of gastric intestinal metaplasia (GIM) and dyspepsia symptoms has not been described. [1, 2] Functional dyspepsia is defined as having at least one of the following symptoms, epigastric pain, postprandial fullness, early satiety, or epigastric burning without a structural cause. [3, 4] The current approach to dyspepsia advocates for an upper endoscopy to be done for those with alarming symptoms or those with new dyspepsia and age 60 or older. [3, 4] The prevalence of recurrent upper abdominal pain and dyspepsia across the U.S. and Western countries is approximately 20–25%, making this clinical entity common in Western societies. [5, 6] Thus, understanding the relationship between gastric intestinal metaplasia to that of functional dyspepsia is fundamentally important, as our current knowledge is quite limited.

Identifying a relationship between gastric intestinal metaplasia and dyspepsia symptoms can provide clarity for an oft functional condition. Moreover, gastric intestinal metaplasia is likely underappreciated in certain groups such as immigrant, ethnic and racial minorities because the burden of Helicobacter Pylori (HP) and GIM is more common in these underrepresented populations. The aim of this study was to describe the findings during upper endoscopy and pathology in the ambulatory setting for patients presenting with dyspepsia compared to those with iron deficiency anemia (IDA). Therefore, our hope was to better understand the rate of gastric intestinal metaplasia among dyspeptic patients in a diverse U.S. patient population.

## METHODS

### Study Population

We developed a cohort based on consecutive upper endoscopic procedures between Jan 1, 2019 and July 30, 2020 for dyspepsia and iron deficiency anemia (IDA) in the San Francisco Health Network (SFHN), an integrated safety-net health system. The San Francisco Health Network (SFHN) included San Francisco General Hospital (SFGH) and community clinics. SFHN is an academic affiliate of the University of California, San Francisco (UCSF). This research study was approved by the UCSF and SFGH Institutional Review Boards (IRB # 20–30255). The SF Health Network primarily delivers clinical care to patients with Medi-Cal, Medicare, Healthy San Francisco (a subsidized health coverage plan) and the uninsured.

### Clinical definitions and data collection

We developed a database using endoscopy, pathology, and laboratory databases paired with chart review of the safety-net population within the San Francisco Health Network. The Rome IV definition of dyspepsia included those patients having at least one of the following symptoms, epigastric abdominal pain, early satiety, postprandial fullness without a structural cause. Patients with reflux and burning symptoms were not included. Patients were electronically referred for an upper endoscopy to gastroenterology for dyspepsia symptoms that occurred over the last year. Patients with alarm symptoms (i.e. weight loss, GI bleeding) were excluded from the cohort. But prior to presenting to endoscopy, GI consultants recommended Helicobacter Pylori testing and treatment, and empiric proton pump inhibitor (PPI) therapy. If they didn’t respond to initial management, they then presented for endoscopy by way of PCP referrals. During the endoscopy, patients with peptic ulcer disease found during endoscopy were excluded. For the IDA group, the group was defined as those patients referred to gastroenterology by primary care physicians with this indication for consideration for endoscopy. The endoscopic procedures were recorded in and extracted from Provation (MD Version 5.0.420.16). Most patients had biopsies done and they were cross reference to the pathological database. The pathological reports were identified, and the pathological diagnosis was extracted from Epic.

### Study outcomes

The primary outcome was the prevalence of gastric intestinal metaplasia in patients with dyspepsia compared to IDA. Secondary outcomes included prevalence of Helicobacter pylori (HP), chronic gastritis (CG) and chronic active gastritis (CAG) in the dyspepsia and IDA groups. Covariates included sociodemographic factors. The sociodemographic factors included race/ethnicity (White, Asian, Black, Hispanic/Latino, Other), gender (female, male), and age (years).

### Statistical Analyses

Analysis was done in Stata (Microsoft, Redmond, WA, USA). Procedures were categorized by cohort dyspepsia and IDA. We summarized sociodemographic factors, EGD findings and pathological findings for dyspepsia compared to IDA. We then used the Rao-Scott Chi-square test to compare demographics to pathological findings for dyspepsia and IDA. We then examined the crude association between each covariate and the binary outcome by utilizing univariate logistic regression models. Lastly, we then performed an adjusted multivariable analysis to assess the independent association between GIM and dyspepsia. P-value less than 0.05 was considered statistically significant.

## RESULTS

### Study Population and Demographic Factors

There were 349 patients identified in the dyspepsia cohort who had undergone an upper endoscopy and 366 patients in the IDA cohort. Among the dyspepsia cohort, the average age was 55 years, 227 (65.0%) female, 53 (15.8%) were White, 128 (38.2%) were Asian, 33 (9.9%) were Black and 125 (35.8%) were Hispanic and 2.9% were other patients. While among the IDA cohort, the average age was 59 years, 173 (47.3%) female, 67 (18.3%) were White, 101 (27.6%) were Asian, 71 (19.4%) were Black and 122 (33.3%) were Hispanic and 1.4% were other patients (Table 1). Overall, compare to IDA, among dyspepsia, patients were younger (55 vs. 59, p < 0.01), female (65% vs. 47%, p < 0.01), Asian (38% vs. 27%, p < 0.01) and Black (9% vs. 19%, p < 0.01).

### Pathology Findings

With respect to pathology findings, compared to patients with IDA, patients with dyspepsia were more likely to harbor gastric IM (20.3% vs. 14.2%, p = 0.03) and less likely to have chronic active gastritis (12.0% vs. 26.5%, p < 0.01) or HP (10.9% vs. 21.3%, p < 0.01) (Table 2). The unadjusted association between IM and dyspepsia was statistically significant (OR 1.5, 95% CI 1.04–2.28, p = 0.03); moreover, the relationship strengthened after adjusting for pathological findings, race, ethnicity, gender and age (adj OR 1.81, 95% CI 1.19–2.76, p < 0.01) (Table 3).

## DISCUSSION

Multiple studies have shown a clear link between peptic ulcer disease and Helicobacter pylori to dyspepsia; but very limited studies have explored the frequency of gastric intestinal metaplasia in functional dyspepsia.[7] In this study, when we compared the presence of GIM in patients with dyspepsia compared to IDA, we found that patients with dyspepsia were more likely to harbor gastric intestinal metaplasia but less likely chronic active gastritis and H. Pylori. In a multivariable analysis, the relationship between gastric intestinal metaplasia and dyspepsia strengthened, suggesting that the symptoms of dyspepsia may be due to these pathological changes. In our study, the increased frequency of GIM in patients with dyspepsia (20.3%) compared to IDA (14.2%) has multiple clinical implications. For one, it can provide an explanation for patients with dyspepsia that the presence of GIM is associated with their symptoms and providing patients an explanatory model. Furthermore, any future interventions that regresses GIM could potentially reduce dyspepsia symptoms. Understanding this relationship between GIM and dyspepsia is valuable, as patients with GIM have an increased number of clinical encounters, more required endoscopies for GIM surveillance, and a higher probability of developing gastric cancer leading to higher morbidity and mortality.

Functional dyspepsia gastroenterology guidelines call for an upper endoscopy to be done for those age 60 and older with new dyspepsia symptoms or anyone with dyspepsia and alarming symptoms.[8] Our study is one of a few in the US that showed a clear association of gastric intestinal metaplasia, often a precursor to gastric cancer, to dyspepsia when compared to IDA.[9] To assess whether the frequency of GIM in patients with dyspepsia was elevated and more likely a true association, we decided to use IDA as a comparator group. An asymptomatic healthy cohort of patients undergoing upper endoscopy was not available for comparison. Instead, we chose patients with IDA because it is a condition that has been associated with Helicobacter pylori and its sequelae (atrophic gastritis, intestinal metaplasia, chronic active gastritis), thereby this often asymptomatic population carries a higher likelihood of GIM. [10] Moreover, we posit that if GIM is more likely to be represented in patients with IDA compared to healthy asymptomatic controls, then an observed increase in GIM in patients with dyspepsia is even more likely to be a true association.

There have been a few non-US studies that explored the incidence of gastric IM in patients with dyspeptic symptoms. The prevalence of IM ranged from 5.9–23.9%, [11–13] which is in the range observed in our study at 20.3%. Moreover, across the world including countries such as Netherlands, Sweden, Iran, Japan and other countries, independent of symptoms, the range of incidence of IM has varied between 3–37%. [12, 14–21] With or without dyspeptic symptoms, these rates are astronomically high.

Thus, multiple countries have conducted surveillance studies describing the regression and progressions rates of intestinal metaplasia. In the Netherlands, a multi-center prospective study showed that among a cohort of patients with IM there was a 32% regression of IM lesions but a 2% progression of IM lesions to more advanced lesions.[21–23] This was independent of dyspepsia symptoms or not. Considering that the rates of IM in the United States range from 7.4–19% and up to 37% in Japan, understanding this effect is important if we expect to reduce the incidence, morbidity and mortality of gastric intestinal metaplasia and its eventual end state, gastric cancer.[19–21, 24] Thus, we hope that our novel work will add to the gastric intestinal metaplasia and dyspepsia national guidelines.

There were several limitations to this study. One limitation is that there is no ideal comparator group since all upper endoscopy procedures were performed for a clinical indication. In patients with IDA, there is a higher prevalence of H. pylori and they are more likely to have an achlorhydria gastric state.[10, 25] We chose this group because they are less likely to be symptomatic; concurrently, the higher prevalence of H. pylori increases the likelihood of GIM thereby making any observed association between GIM and dyspepsia more likely to be a true association. Another limitation is that among dyspeptic patients, we included a constellation of symptoms which is reflective of the varied presentation of this condition. Another limitation is the potential for residual confounders such as diabetes and other comorbidities and their effect on the primary outcome. Regardless of these limitations, this study is one of a few US studies exploring the incidence of IM among dyspeptic patients.

## CONCLUSION

In conclusion, this study showed a novel relationship between the presence of gastric intestinal metaplasia and dyspepsia symptoms. We demonstrated that after adjusting for confounding factors, the relationship strengthened, suggesting a possible casual role for gastric IM in dyspepsia. Future studies should not only verify the relationship between GIM and dyspepsia, but explore subtypes of dyspepsia symptoms and their relationship with GIM. Other than treating H. pylori, reducing smoking and alcohol intake, interventions (i.e. two-endoscope technique of endoscopic mucosal resection, EMR) that modulate or reverse GIM should be tested to see if the intervention will reduce symptoms of dyspepsia.[26, 27] Overall, this study represents one of a few US studies to clearly show an increased association of gastric intestinal metaplasia among patients with dyspepsia.

## Figures and Tables

**Table 1:** Demographics of patients with dyspepsia and iron deficiency anemia who underwent an endoscopy

Demographics	n (%) – Dyspepsia	n (%) – IDA	P-value
Number	349 (100)	366 (100)	
Gender			
Female	227 (65.0)	173 (47.3)	< 0.01
Male	122 (35.0)	193 (52.7)	< 0.01
Race/Ethnicity			
White	53 (15.8)	67 (18.3)	0.26
Asian	128 (38.2)	101 (27.6)	< 0.01
Black	33 (9.9)	71 (19.4)	< 0.01
Hispanic/Latino	125 (35.8)	122 (33.3)	0.49
Other	10 (2.9)	5 (1.4)	0.16
Age, median (range)	55.1 (17–82)	59.9 (29–89)	< 0.01

* Abbreviations: IDA, iron deficiency anemia

**Table 2:** Pathological Findings in patients with Dyspepsia and Iron Deficiency Anemia

Pathological Findings*	n (%), Dyspepsia	n (%), IDA	P score
Chronic gastritis	40 (11.5)	32 (8.7)	0.23
Chronic active gastritis	42 (12.0)	97 (26.5)	< 0.01
Helicobacter pylori	38 (10.9)	78 (21.3)	< 0.01
Intestinal metaplasia	71 (20.3)	52 (14.2)	0.03

**Table 3:** Unadjusted and adjusted analyses of factors associated with dyspepsia compared to iron deficiency anemia

Pathological Findings	Unadjusted OR (95% CI)*	P-value	Adjusted OR (95% CI)**	P-value
Intestinal Metaplasia (IM)	1.54 (1.04–2.28)	0.03	1.81 (1.19–2.76)	< 0.01
H. Pylori (HP)	0.45 (0.29–0.69)	< 0.01	0.98 (0.50–1.92)	0.96
Chronic Active Gastritis (CAG)	0.38 (0.25–0.56)	< 0.01	0.35 (0.18–0.67)	< 0.01
Chronic Gastritis (CG)	1.35 (0.83–2.21)	0.23	0.93 (0.54–1.58)	0.56

* Reference group is iron deficiency anemia

** Adjusted for path findings, race/ethnicity, gender, and age

## Data Availability

The datasets used during the current study are available from the corresponding author on reasonable request.

## STUDY HIGHLIGHTS

### WHAT IS KNOWN

- Evidence exist for the role of peptic ulcer disease (PUD) and Helicobacter pylori (HP) in dyspepsia, though the role of gastric intestinal metaplasia (IM) in dyspepsia is not known.

### WHAT IS NEW HERE

- We observed a significant relationship between the presence of gastric IM and dyspepsia symptoms.
- After adjusting for confounding factors, the relationship strengthened, suggesting a possible casual role for gastric IM in dyspepsia.

## Abbreviations

PUD: Peptic ulcer disease
HP: Helicobacter Pylori
U.S.: United States
GIM: Gastric Intestinal Metaplasia
CG: chronic gastritis
CAG: chronic active gastritis
IDA: iron deficiency anemia
